# Prevalence of preconception risk factors for adverse pregnancy outcome among women from tribal and non-tribal blocks in Nashik district, India: a cross-sectional study

**DOI:** 10.1186/s12978-022-01473-z

**Published:** 2022-07-27

**Authors:** Prakash Prabhakarrao Doke, Jayashree Sachin Gothankar, Amruta Paresh Chutke, Sonali Hemant Palkar, Archana Vasantrao Patil, Prasad Dnyandeo Pore, Khanindra Kumar Bhuyan, Madhusudan Vamanrao Karnataki, Aniruddha Vinayakrao Deshpande, Aparna Nishikant Shrotri, Arvinder Pal Singh Narula

**Affiliations:** 1 Department of Community Medicine, Bharati Vidyapeeth Deemed University Medical College, Pune, 411043 India; 2 State Family Welfare Bureau, Department of Public Health, Government of Maharashtra, Pune, 411001 India; 3 UNICEF, Maharashtra, Mumbai India

**Keywords:** Married women desiring pregnancy, Preconception risk, Prevalence, Tribal area, Undernutrition, BMI

## Abstract

**Background:**

Although critical, the preconception phase in women’s lives is comparatively ignored. The presence of some risk factors during this phase adversely affects the wellbeing of the woman and the pregnancy outcome. The study objectives were to measure the prevalence of various known risk factors for adverse pregnancy outcome in the preconception period of women and their comparison between blocks.

**Methods:**

This was a community-based cross-sectional study in two tribal and two non-tribal blocks each in Nasik district, Maharashtra, India. The study included married women desiring to conceive within 1 year. Trained Accredited Social Health Activists (field level health worker) collected information from women using a validated interview schedule through house-to-house visits and obtained women’s anthropometric measurements in a standard manner. The study assessed the presence of 12 documented risk factors.

**Results:**

The study enlisted 7875 women desiring pregnancy soon. The mean age of women was 23.19 (± 3.71) years, and 16% of them were adolescents. Women’s illiteracy was higher in tribal areas than non-tribal (p < 0.001). About two-thirds of women have at least one risk factor, and 40.0% have a single risk factor. The most common risk factor observed was no formal education (44.35%). The prevalence of selected risk factors was significantly higher among women from tribal areas. The mean BMI of women was 19.73 (± 3.51), and a higher proportion (40.5%) of women from tribal areas had BMI < 18.5. Despite being of high parity status (≥ 4), about 7.7% of women from the tribal area and 3% from non-tribal desired pregnancy. Tobacco and alcohol consumption was higher among tribal women. The majority of women consumed meals with family members or husbands. Protein and calorie intake of about 1.4% of women was less than 50% of the recommended daily allowance; however, most of them perceived to have abundant food.

**Conclusions:**

Health risks, namely younger age, illiteracy, high parity, consumption of tobacco, low protein, and calorie intake, were quite prevalent, and the risks were significantly more among women from tribal areas. “Continuum of care” must comprise preconception care inclusive of Behavioral Change Communication, particularly for easily modifiable risk factors and specially for tribal women.

## Plain language summary

Women’s health during the preconception phase although important, is an ignored period in her life cycle. Literature has shown that the presence of risk factors in women during the preconception phase is hazardous to the health of women and newborns. The present study is a cross-sectional study conducted in four blocks of Nasik district, Maharashtra, India, to measure risk factors for adverse pregnancy outcome among women and its comparison between blocks.

We included married women desiring conception within 1 year. Accredited Social Health Activists asked questions using a validated interview schedule and recorded women’s anthropometric measures.

Of the 7875 women, 16% were adolescents, and the mean age of women was 23.19 ± 3.71 years. About two-fifth of women had one risk factor, the commonest being no formal education. Overall mean BMI of women was 19.73 (± 3.51). The prevalence of risk factors was significantly higher among women from tribal areas. Despite having ≥ 4 parity a higher proportion of women from tribal areas desired to conceive. About 1.4% of women had protein and calorie intake below 50% recommended consumption.

In conclusion, the prevalence of selected risk factors was significantly higher among tribal women. The study identifies the need for preconception care services.

## Introduction

### Background

Women’s health throughout their childbearing age should be addressed to improve obstetrical and perinatal outcomes [1]. Preconception health refers to the health and wellbeing of persons of the reproductive age group before pregnancy. Overall health during the pre-conceptional phase is critical because it influences reproductive health, fertility, maternal and neonatal outcomes, and future generations’ health [2]. The period around conception (2–3 months before and after) is crucial for optimizing gamete function and early placental development. Crucial steps in organogenesis occur early in fetal development [3]. The presence of risk factors during this phase increases the risk of adverse outcomes of pregnancy. Various studies have shown a strong relationship between women’s overall health or risk factors before pregnancy and maternal and child health outcomes [4, 5].

The ten comprehensive recommendations from Center for Disease Control and Prevention to improve women’s health and thereby pregnancy outcome include attention to recognized risk factors, their management, and preventive measures [6]. Among risk factors of non-communicable diseases, consumption of alcohol/tobacco and nutritional status are crucial. The risk factors like malnutrition, multiparity can be assessed through a simple interview schedule, while very few risk factors like anemia need laboratory investigation for their identification. Most of the risk factors are amenable to correction hence labelled as modifiable. These risk factors also vary among countries and in different communities. In countries like India, women and adolescent girls eat last and generally consume inadequate food due to cultural and gender norms; these practices may have long-term adverse outcomes for their children [7].

Many risk factors and intervention areas have been identified [4]. The women certainly have health risks if their medical, social, environmental, physical, financial, educational, or other socioeconomic factors are considered hazardous [8]. Many studies present women's nutrition or chronic non-communicable disease aspects, while many are retrospective interviews of pregnant women or delivered women. Most of the reported studies are hospital-based, emphasizing the effectiveness of preconception care, management of chronic diseases, diabetes control, and folic acid supplementation [9–11]. Similarly, many reports are analyses of system-generated data, particularly from Pregnancy Risk Assessment Monitoring in the USA (PRAMS). However, studies focusing exclusively on women's health and identifying risk factors in the preconception phase are rare in India.

Women having risk factors need both population and individual level support to reduce risks and plan pregnancy. Besides, women requiring preconception health promotion are more likely to have poor education, low income, poor health status before or during pregnancy. These may lead to adverse maternal, fetal, and childhood health [12]. The women either do not seek prenatal care or seek after conception, which may be too late. Knowing risk factors among women desiring pregnancy is essential for planning local specific policies.

World Health Organization has recommended the implementation of preconception in all countries. Accordingly, preconception care (PCC) intervention has been planned and implemented in the Peint and Sinnar blocks of the Nashik district. The outcomes are measured and compared with comparison blocks. The project comprised four stages; identifying women desiring conception within a year, assessing their health status, especially risk factors for adverse pregnancy outcome, providing preconception care, and comparing outcomes with the control group. The present community-based cross-sectional study describes the first two stages.

### Objectives

1. to measure the prevalence of various risks factors for adverse pregnancy outcome among women in the preconception period and
2. to compare the risks between study and comparison blocks and tribal and non-tribal blocks.

## Material and methods

### Study design

A cross-sectional study wherein women of the reproductive age group desiring pregnancy within one year were interviewed and further interrogated. This study was an initial phase of an large intervention study.

### Setting

**Fig. 1:**
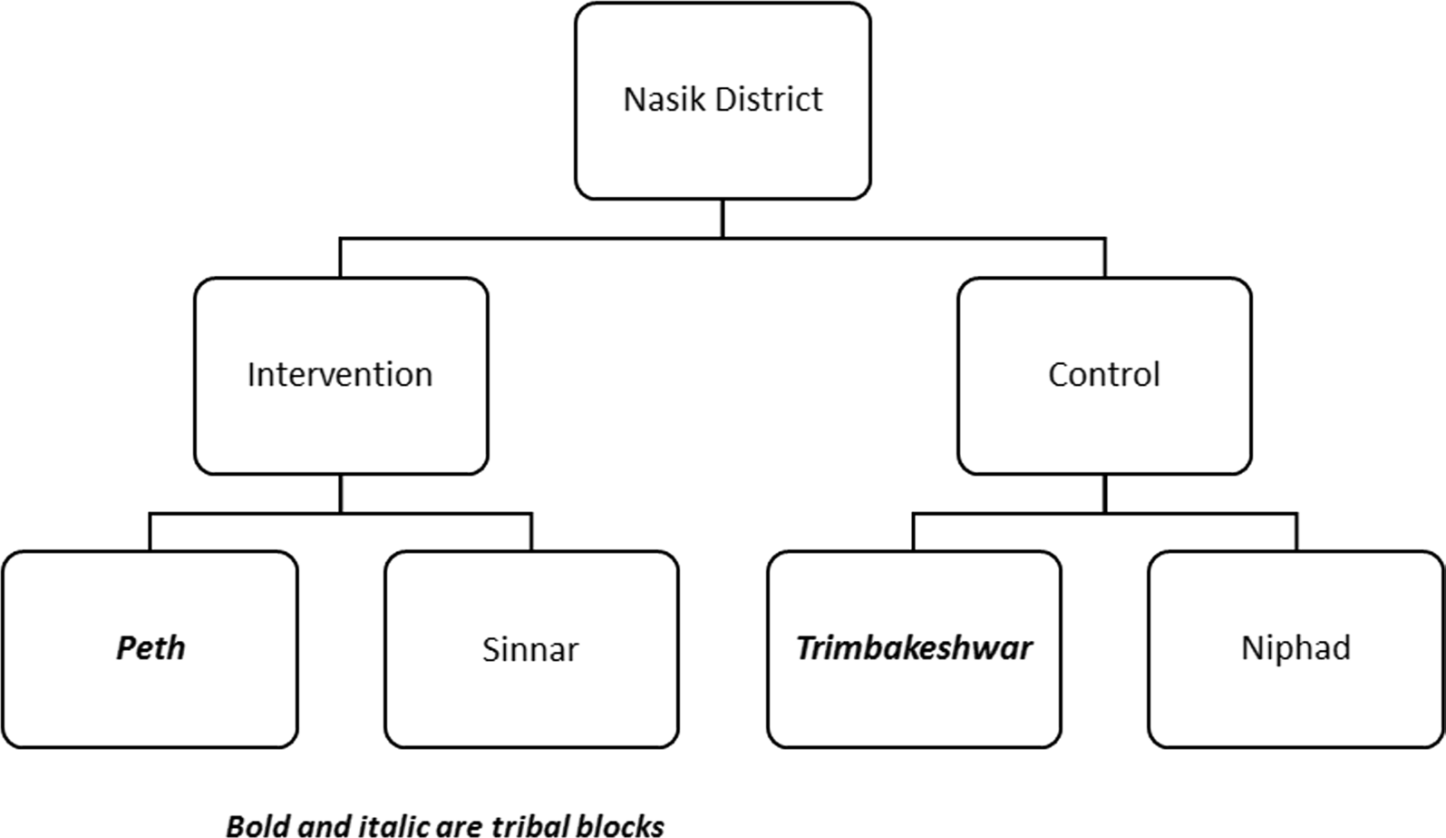
Blocks in intervention and control arm of the study, Nashik District, 2018–19

**Fig. 2:**
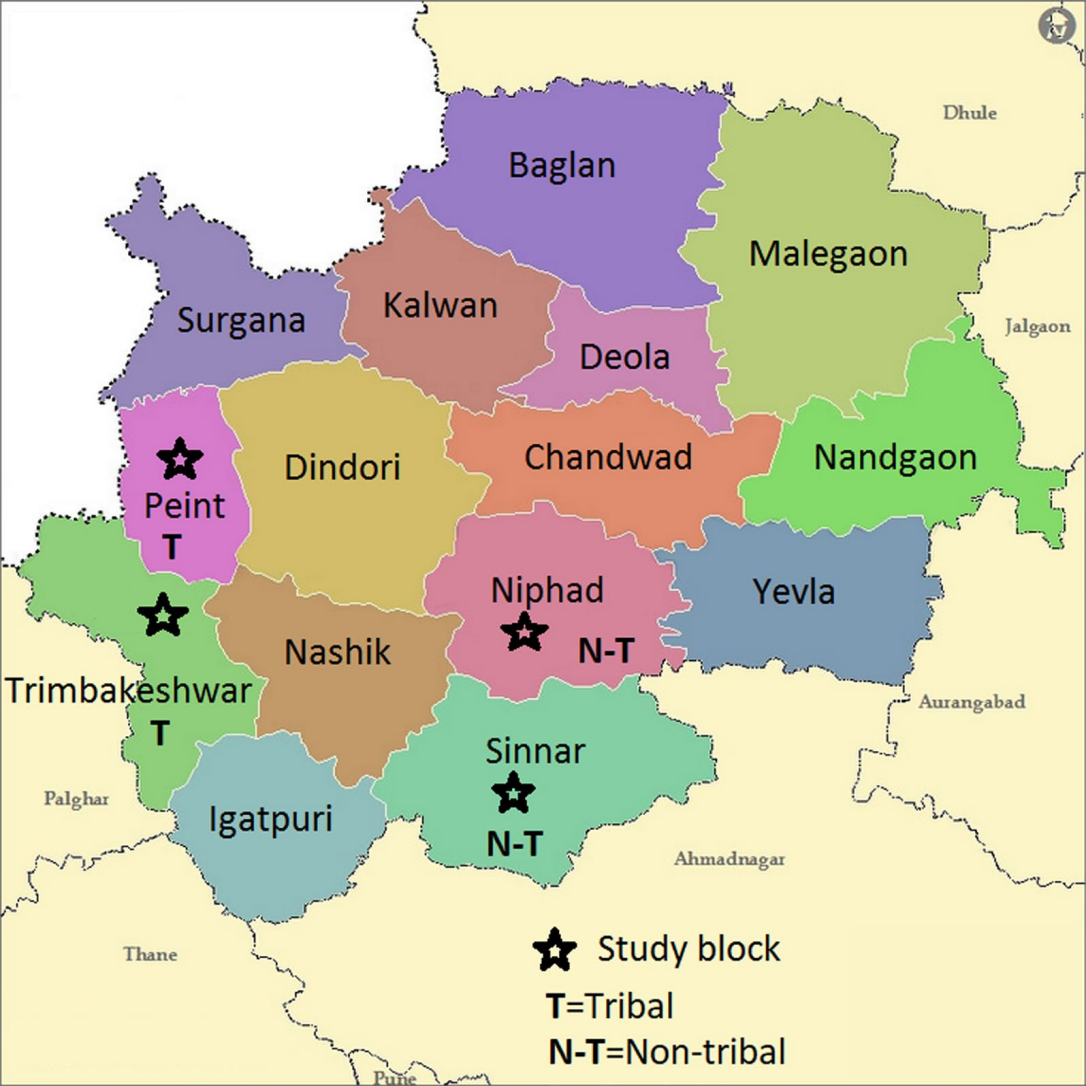
Geographical location of study blocks, Nashik District

The authors conducted this community-based study in the rural population of Nashik district, Maharashtra, India. In the district, about 57.5% population is rural, inclusive of 25.6% tribal. Maharashtra government had notified nine blocks out of 15 and 52 Primary Health Centers (PHCs) out of 106 as tribal. Government notifies a block as tribal if more than 50% population is scheduled tribe (ST) and district as tribal if the scheduled tribe population is more than 0.2 million (Based on Maharashtra state 1981 census data). We randomly selected one tribal (Peint) and one non-tribal (Sinnar) block for proposed intervention, and one adjacent tribal (Trimbakeshwar) plus one adjacent non-tribal (Niphad) block was purposively assigned for comparison (Fig. 1). Figure 2 gives the geographical location of these blocks. The study area covered 28 PHCs. As per the last census, the total population of these four blocks was 1,127,902 [13].

The study period was from April to December 2018. Actual enlisting and assessing desiring women were completed in 2 months, July to August 2018.

Specially trained Accredited Social Health Activists (ASHAs) collected the data through the house-to-house visit. ASHAs deposited the completed forms at the respective PHC, PHC sent the forms to the nodal institution fortnightly. The authors created a system of a unique identification code for each participant. Before data entry, the centrally trained supervisory staff reviewed and verified the forms for completeness, legibility, and accuracy. The operator concurrently carried out data cleaning, validity, and data entry.

### Participants

**Fig. 3:**
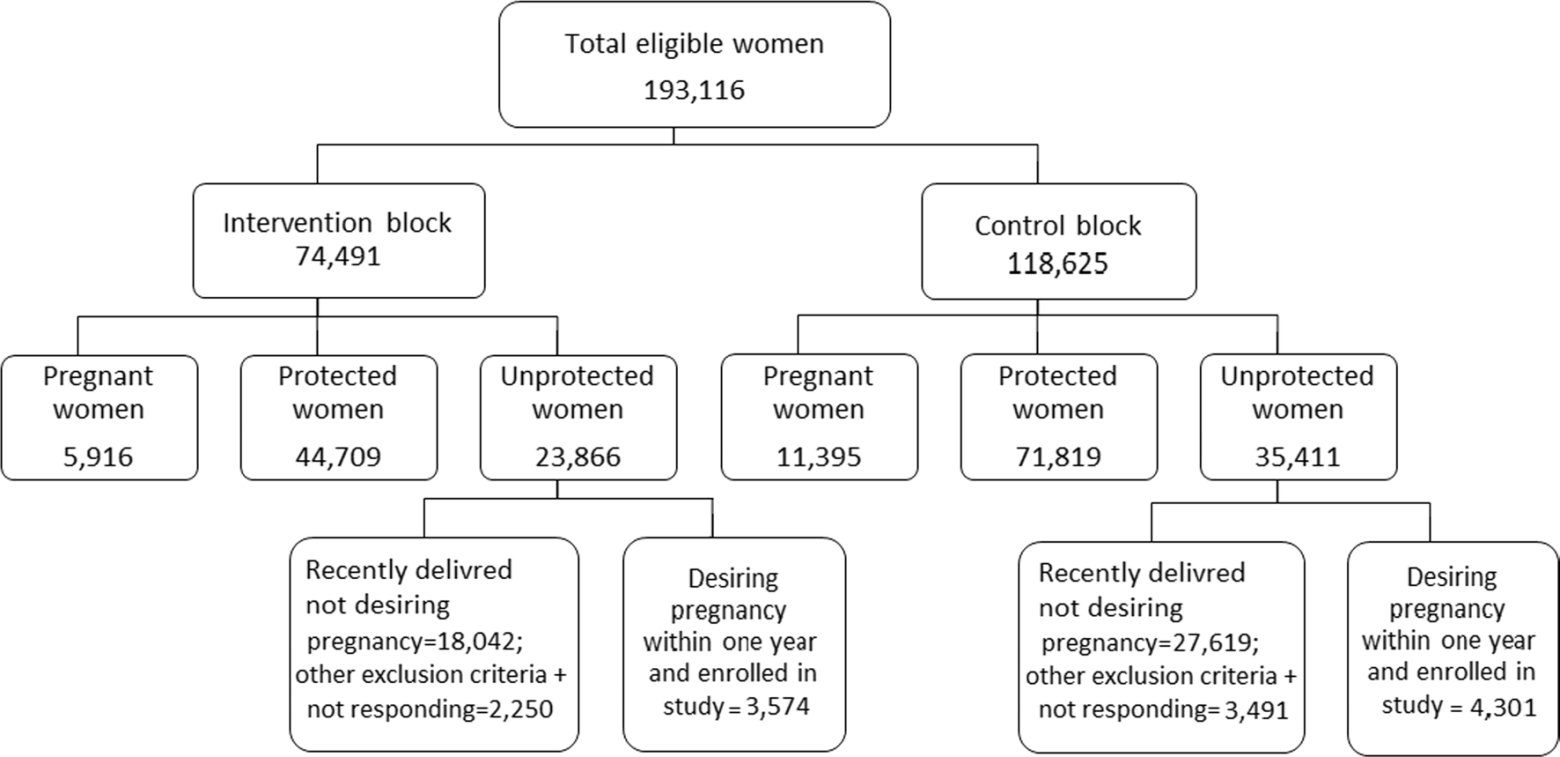
Flow diagram of women in the study, Nasik District, 2018–19

Currently, married women in the reproductive age group and desiring to conceive within 1 year, residing in the area (or intend to live for more than 6 months) were included and enrolled in the study. We excluded the women unable to understand Marathi, Hindi, or English and unable to respond due to psychiatric illness (Fig. 3).

### Variables

ASHAs collected information about occupation, religion/caste, and type of family. Authors considered the following 12 risk factors; age less than 20, age more than 35, illiteracy, multiparity, consanguineous marriage, BMI less than 18.5, BMI more than 25, calorie intake less than 50% of recommended daily allowance, protein intake less than 50% of recommended daily allowance, tobacco consumption in any form [smoking, eating, *Mishri* (roasted tobacco application), snuff and passive smoking], alcohol consumption and adverse outcome of last pregnancy (abortion or stillbirth). We considered the nutritional status using the World Health Organization’s standards [14]; and calculated the percentage of underweight women (BMI < 18.5), normal weight (BMI = 18.5 < 25.0), overweight (BMI ≥ 25 < 30), obese (BMI ≥ 30). Information regarding parity and history of consanguinity were parts of obstetric history. The authors used the 24-h dietary recall method to calculate energy and protein intake. We considered intake of less than 50% of the recommendation as a risk factor for analysis. ASHAs asked practice of taking the meal, type of food, and perception about the adequacy of food consumption.

### Data sources and measurements

The authors prepared the interview schedule, pretested it after validation from experts. Language expert translated the final tool in the local language, Marathi, and back-translated. The interview schedule included socio-demographic variables, obstetric history, nutritional information, and a physical assessment like height, weight, and dietary recall. ASHAs measured the height and weight of the women using standard equipment at home or Anganwadi or the nearest health facility (sub-center or PHC). They recorded height to the nearest 0.1 cm and weight nearest 0.1 kg. The ASHAs weighed the women with light clothes without footwear.

### Sample size and sampling

Amongst the documented prevalence of selected risk factors alcohol consumption had the lowest prevalence of 0.7% [15]. The estimated sample size considering this prevalence of alcohol consumption with an absolute difference of 0.2% with a 95% confidence interval was 6,951 women. A Health Management Information System (2017–18) indicated that the desired sample size would be undoubtedly available in these four blocks. We included all women willing to provide information.

### Ethical considerations

We obtained the Institutional Ethics Committee approval before the initiation of the study. ASHAs obtained written informed consent from all participants before enrolment.

### Statistical methods

Data were coded, anonymized, and analyzed using the Statistical Package for Social Sciences (SPSS) software 25.0 version. The authors have presented the study findings as absolute numbers, proportions, and chi-square tests are applied. As data pertains to a large sample, we decided a p-value less than 0.01 as significant.

## Results

In the study of 7,875 women participated. Overall missing data was less than 15%, excepting caste. Out of the total women, 37.8% belonged to tribal blocks. The majority (88.6%) were Hindu. The minimum age of desiring women was 14.42 years, and the maximum age was 45.50 years (mean 23.19 ± 3.71). Table 1 gives the socioeconomic information about occupation, caste and type of family of the participating women. The distributions of all three socio-demographic demographic variables between four blocks were different; however, the chi-square values were many folds for differences among tribal and non-tribal populations. Overall, the most common occupation among women was farming (48.0%). The highest formally unemployed women belonged to non-tribal blocks (45.8%). Joint families are more common in non-tribal blocks.

**Table 1:** Socio-demographic characteristics of women in Nashik District, India, 2018–19

Characteristics	Study n = 3574 (%)	Control n = 4301 (%)	χ^2^ (p)	Tribal n = 2975 (%)	Non-tribal n = 4900 (%)	Total N = 7875 (%)	χ^2^ (p)
Occupation (n = 7364)							
Professional	36 (1.0)	97 (2.3)	197.17 (< 0.001)	15 (0.5)	118 (2.4)	133 (1.7)	1225.03 (< 0.001)
Semi professional	13 (0.4)	12 (0.3)		2 (0.0)	23 (0.5)	25 (0.3)	
Clerical	3 (0.1)	17 (0.4)		2 (0.0)	18 (0.4)	20 (0.3)	
Shop Owner	57 (1.6)	89 (2.1)		30 (1.0)	116 (2.4)	146 (1.9)	
Farmer	2032 (56.9)	1747 (40.6)		2103 (70.7)	1676 (34.2)	3779 (48.0)	
Skilled worker	77 (2.2)	125 (2.9)		19 (0.6)	183 (3.7)	202 (2.6)	
Semiskilled worker	42 (1.2)	54 (1.3)		27 (0.9)	69 (1.4)	96 (1.2)	
Unskilled worker	110 (3.1)	160 (3.7)		53 (1.8)	217 (4.4)	270 (3.4)	
Unemployed	1027 (28.7)	1666 (38.7)		449 (15.1)	2244 (45.8)	2693 (34.2)	
Caste (n = 6437)							
Scheduled Caste	495 (13.9)	804 (18.7)	160.4 (< 0.001)	556 (18.7)	743 (15.2)	1299 (16.5)	2127.42 (< 0.001)
Scheduled Tribe	1493 (41.8)	1097 (25.5)		1716 (57.7)	874 (17.8)	2590 (32.9)	
OBC	3 (0.1)	10 (0.2)		1 (0.0)	12 (0.2)	13 (0.2)	
Open	462 (12.9)	546 (12.7)		65 (2.2)	943 (19.2)	1008 (12.8)	
Other	675 (18.9)	852 (19.8)		48 (1.6)	1479 (30.2)	1527 (19.4)	
Type of family (n = 6990)							
Nuclear	446 (12.5)	559 (13.0)	13.32 (< 0.001)	399 (13.4)	606 (12.4)	1005 (12.8)	43.81 (< 0.001)
Joint	2723 (76.2)	3128 (72.1)		2117 (71.2)	3734 (76.2)	5851 (74.3)	
Other	42 (1.2)	92 (2.1)		14 (0.5)	120 (2.4)	134 (1.7)	

**Fig. 4:**
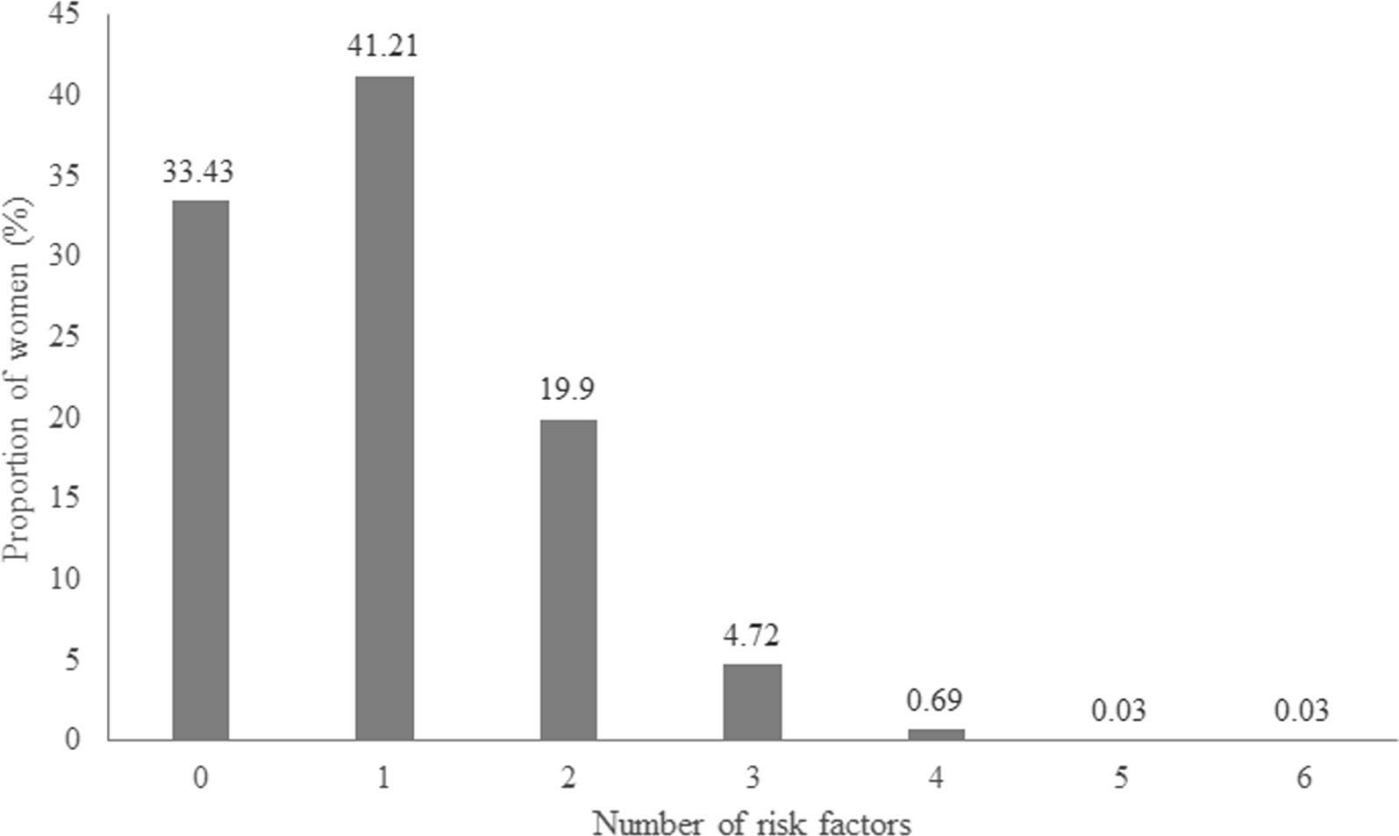
Proportion of women with identified risk factors

About two-thirds of women had some risk factor. About 40% of women had only one risk factor. The remaining women had two or more risk factors. Figure 4 gives the details of women having several risk factors. Among women having only one risk factor, the commonest was no formal education (44.35%), followed by calorie intake less than 50% of daily recommended and age less than 20 years (13.68%). Only two women had five risk factors, and two women had six.

The prevalence of various risk factors and their comparison are given in Table 2. The chi-square values for differences between women from tribal and non-tribal areas were glaringly higher than for differences between intervention and comparison blocks. Amongst the type of tobacco consumption, snuff (inhalation of roasted tobacco powder) was exclusively observed among women from tribal areas (1.5%). Mishri (roasted and powdered tobacco applied to gums/teeth) was the most common tobacco addiction in all blocks together (2.7%).

**Table 2:** Risk factors among women during preconception phase, Nashik, India, 2017–18

S. No	Risk factor	Study n = 3574	Control n = 4301	χ^2^ (p)	Tribal n = 2975	Non-tribal n = 4900	χ^2^ (p)
1	Age ≤ 19 years (n = 7783)	602	654	3.03 (0.08)	561	695	32.03 (< 0.001)
2	Age ≥ 35 years (n = 7783)	58	56	3.25 (0.07)	32	41	1.22 (0.27)
3	Illiterate (n = 7482)	325	408	0.70 (0.40)	490	243	327.26 (< 0.001)
4	Consanguinity (n = 7137)	613	879	20.36 (< 0.001)	592	900	7.08 (0.008)
5	Multiparity ≥ 4 (n = 7647)	48	58	0.001 (0.98)	76	30	52.94 (< 0.001)
6	Tobacco consumption (n = 7804)	205	219	1.84 (0.17)	312	112	247.3 (< 0.001)
7	Alcohol consumption (n = 7804)	18	30	1.14 (0.29)	30	18	12.75 (< 0.001)
8	Less calorie intake (n = 7872)	40	71	3.97 (0.046)	40	71	0.14 (0.70)
9	Less protein intake (n = 7871)	33	71	7.93 (0.01)	16	88	22.49 (< 0.001)
10	BMI < 18.5 (n = 7169)	1355	1400	13.57 (< 0.001)	1206	1549	114.69 (< 0.001)
11	BMI ≥ 25 (n = 7169)	167	273	13.55 (< 0.001)	92	348	46.81 (< 0.001)
12	Previous adverse outcome (n = 3656)	81	106	0.762 (0.38)	70	117	0.01 (0.94)

Table 3 gives information about meal practices, perceptions about consumption food quantity, and type of food. The majority of the women perceived that they consume abundant food (64.5%). Women from tribal areas consumed more non-vegetarian food than women from non-tribal areas.

**Table 3:** Food habits of women during preconception phase Nashik, India, 2017–18

Food habits	Study n = 3574 (%)	Control n = 4301 (%)	χ^2^ (p)	Tribal n = 2975 (%)	Non-tribal n = 4900 (%)	Total N = 7875 (%)	χ^2^ (p)
Meal Consumption practice (n = 7052)							
With husband	568 (15.9)	816 (19.0)	33.29 (< 0.001)	577 (19.4)	807 (16.5)	1384 (17.6)	49.67 (< 0.001)
With family members	2628 (73.5)	2861 (66.5)		1943 (65.3)	3546 (72.4)	5489 (69.7)	
After men	43 (1.2)	87 (2.0)		17 (0.6)	113 (2.3)	130 (1.7)	
Last	29 (0.8)	20 (0.5)		18 (0.6)	31 (0.6)	49 (0.5)	
Perception of women regarding food quantity consumed on an average (n = 6995)							
Abundant	2358 (66.0)	2724 (63.3)	10.42 (0.015)	1883 (63.3)	3199 (65.3)	5082 (64.5)	12.23 (0.007)
Enough	848 (23.7)	956 (22.2)		597 (20.1)	1207 (24.6)	1804 (22.9)	
Less	36 (1.0)	56 (1.3)		38 (1.3)	54 (1.1)	92 (1.2)	
Remaining	2 (0.1)	15 (0.3)		9 (0.3)	8 (0.2)	17 (0.2)	
Type of food (n = 7158)							
Vegetarian	1598 (44.7)	1737 (40.4)	46.46 (< 0.001)	771 (25.9)	2564 (52.3)	3335 (42.3)	487.36 (< 0.001)
Non-vegetarian	602 (16.8)	952 (22.1)		721 (24.2)	833 (17.0)	1554 (19.7)	
Occasional non-veg	1119 (31.3)	1150 (26.7)		1122 (37.7)	1147 (23.4)	2269 (28.8)	

ASHAs measured the height and weight of 7167 (91.0%) women. The Mean BMI of women was 19.73 (± 3.51). The mean protein and caloric intake were 41.69 (+ 8.01) gm. and 1666.92 (± 278.37) kcals. None of the women in the BMI > 30 perceived that they were eating lesser/leftover food. A majority, i.e., 70.0%, of undernourished women perceived that they consume abundant food.

## Discussion

The present community-based study is more extensive than several studies. It was observed that most of the variables were statistically significant between the intervention and control groups as well as between tribal and non-tribal groups (p < 0.01) (Tables 1 and 2). This statistical significance was observed sheer due to the large sample size. However, the chi-square values for the difference between tribal and non-tribal groups were many-fold of the value between study and control groups. Women residing in tribal and non-tribal areas differ substantively.

In India, due to cultural norms, most married women, especially in rural areas, are expected to remain pregnant soon after marriage. Although critical to her health, the period after the wedding and before the pregnancy is usually ignored. Most of the women become pregnant within 1 year of marriage. Often, women who get married below 18 years of age tend to have an unplanned pregnancy and are most likely to be anemic and malnourished at conception and likely to have a miscarriage or stillbirth [16]. The concept of a "continuum of care" for improving pregnancy outcomes and reducing maternal and neonatal mortality implies the need for preconception care [17]. However, a wide gap exists in the continuum of care because of the focus only on pregnancy care and almost indifference towards preconception care. It is acknowledged that the preconception period is the most critical. To acknowledge its importance, we need to include preconception in the continuum of care and rename the program as Reproductive, Preconception, Maternal Neonatal Child Health and Adolescents (RPMNCHA).

In the USA, the Behavioral Risk Factor Surveillance System (BRFSS) and PRAMS include preconception risk factors and publish the reports regularly. The known risk factors included in these reports can be grouped into socio-demographic, diseases based on examinations/investigations, and service components. The number of risk factors considered in various studies ranged from 5 to 14 [10, 11, 18–22]. All studies have considered smoking as a risk factor. Almost all considered alcohol consumption, BMI outside normal weight range, unintended pregnancy, non-communicable diseases, and absence of preconception care. A study in Italy identified 11 risk factors, including social, and did not use any laboratory investigations for assessment like the present study; but included, risk factors like citizenship, married status, etc., which were not considered in the present study [10]. A large study analyzing selected five risk factors observed that 52% of women had some risk factor (18.7% had multiple risk factors, 33.3% had only one risk factor), and 48.0% had none [22]. The present study observed about two-third of women has some risk factor. In contrast, a study in a district adjacent considered 22 risk factors and revealed that all women had at least one risk factor [11].

### Teenage pregnancy

The problem is typical of India and other Low and Middle-Income Countries (LMICs). As per national-level surveys for Maharashtra, 33.7% of women began childbearing at 18 or 19 years [15]. Despite being pregnant, around 34.5% of women do not undergo any checkup in the first 3 months of the pregnancy [15]. Most of the fetal organs have been formed when women visit the health care system for pregnancy care. This fact highlights that to improve the quality outcome of current and subsequent pregnancy, women who plan to conceive should seek preconception care [17].

The current study reported that 15.9% of married women below the age of 19 were desirous of becoming pregnant within the following year. The proportion was higher among women from the tribal area (p < 0.001). Another study conducted in a Municipal Corporation area in an adjacent district identified that teenage pregnancy was 18% [11]. This age distribution is similar to population-based studies conducted in other parts of India [17, 23–25]. Marriage at a younger age leading to early conception is a health risk significantly associated with postnatal complications, other adverse pregnancy outcomes, and high chances of unplanned pregnancies [26, 27]. Social pressure to marry early and for early childbearing soon after marriage often prevents these married women and young girls from accessing contraception [27]. The educational opportunities and better socioeconomic status in non-tribal areas might be the reasons for the lower proportion of teenage pregnancies in non-tribal parts. Illiteracy can be seen as a social health risk for women; it was reported more in tribal than non-tribal women (p < 0.001). At least two studies also considered lower education as a risk factor [10, 11].

### Multiparity

The present study reports that about 5% of women desire pregnancy in the next year despite giving birth to three or more children. The difference was statistically significant between tribal and non-tribal areas (p < 0.001). This highlights the scope for extending family planning services and the importance of interventions that may reduce unintentional births [28, 29].

### Tobacco consumption

Smoking in the preconception period is associated with delayed conception and infertility [2]. Convincing studies about the effect of smoking during the preconception period are lacking, except one showing a higher risk of gastroschisis and omphalocele [28]. However, indirect evidence of impact at the population level is evident from introducing smoke-free legislation in different countries associated with significant reductions in preterm births [4]. In the USA, 18.5% of non-pregnant and not undergone hysterectomy women smoke [22]. The meta-analysis results reported that maternal active and passive smoking is associated with a higher risk of congenital heart disease among the offspring [28, 30]. In the current study, however, the prevalence of smoking was low, i.e., 1.3% of the women in tribal blocks were smokers. But the prevalence of consumption of tobacco in any form was 3.8%. Tobacco use was significantly more in women from the tribal area than non-tribal (p < 0.001). Mishri use is widespread in the tribal area. One Indian secondary analysis study among women in the reproductive age group reports slightly higher tobacco use rates of 11% [31].

### Alcohol consumption

Maternal alcohol consumption leads to various fetal alcohol spectrum disorders and a 30% increase in spontaneous abortion [20]. Since many pregnancies are unplanned, the scope for action at the individual level is limited, highlighting the importance of cost-effective public health actions to reduce risk behaviors in the whole population. The safest approach is to abstain from alcohol when planning a pregnancy and during pregnancy [2, 4]. In the current study, the reported alcohol consumption was meager among the women, i.e., 0.7% compared to other studies [9, 20] and particularly from the USA (16%) [19]. In European countries, the proportion may be as high as 70% [18]. Smoking, alcohol consumption, and refusal of HIV testing are considered important risk factors, and the overall proportion of any of the three risk factors has been reported as more than 50% [19].

### BMI

In western countries, obesity is the most common preconception risk factor [22]. In the current study, almost one-third of women were undernourished (BMI < 18.5), similar to other studies [24, 32, 33]. In the tribal block, the proportion was more than 40% proportionate to their calorie and protein intake. Overall, undernutrition was more, and overweight was less in the present study than observations in the NFHS 5 report [34]. There could be three reasons; our data is about 3 years later; secondly, the population is exclusively women desiring pregnancy in the immediate future; and the population consisted of a substantial number of women from tribal areas. The high prevalence of underweight among pre-pregnant women may not be attributed to discrimination against young women. Most women consumed food with other family members or husbands in the current study. Undernutrition can be attributed to other factors like exposure to infectious disease, poor sanitation, and poor diets, contributing to women's low body mass. Interestingly, irrespective of other factors, the women in this study are satisfied with their food consumption practices. Surprisingly, although most women perceived that they consumed adequate or abundant food consumption, many were undernourished. Undernutrition needs to be addressed before becoming pregnant to improve their pregnancy outcome.

Studies reported an association between pre-pregnancy BMI and the risk of adverse perinatal outcomes, i.e., infants whose mothers were underweight had a significantly higher risk of being small for gestational age, low birth weight, or the possibility of preterm birth [33–39]. All women require appropriate pre-pregnancy advice and interventions to optimize BMI, especially in adolescents. [36, 38].

A study in Asia did not find a significant association between low pre-pregnancy BMI and preterm birth; the reason may be the lack of well-conducted cohort studies [40].

The authors used the 24-h dietary recall method to assess dietary intake for two reasons; its feasibility and almost no loss to recall memory. This method is most commonly used for nutritional surveys [41].

One study from adjacent state of Karnataka reported the proportion of women consuming less than 50% of the RDA was 15.8% for energy, 39.6% for protein [24], which contrasts with the current study, where only 1.4% of the women consumed less than 50% of their daily energy and protein requirements. At the same time, almost half of the women were consuming 70% or more of their recommended energy intake per day.

### Consanguinity

Only two studies considered consanguineous marriage as a risk factor, and prevalence was 2.9 and 20% [11, 18]. In a study in North Karnataka, India, 26.9% of women had a history of consanguineous marriage, while the current study reported it to be 19%. Consanguinity is associated with adverse perinatal outcomes, pregnancy loss, stillbirths, and low birth weight babies [42]. Out of 12 risk factors BMI is dependent on calorie and protein intake. BMI, tobacco, and alcohol consumption are directly behavior-related. Individual Behavior Change Communication can address these risk factors. In addition, community-level Behavior Change Communication can modify adolescent pregnancy, multiparity, consanguinity and illiteracy. Once risk factors are modified subsequent outcomes will surely improve.

### Limitations

The 24-h dietary recall method doesn't capture weekly variations (usually substantial) and had difficulty precisely estimating the intake quantity. The missing data was high. We did not conduct laboratory investigations to support the assessment of risk factors. We did not analyze tribal and non-tribal women-wise.

## Conclusions

Health risks, namely younger age, illiteracy, farming with moderate to high-intensity work, high parity, low consumption of protein and calories, and undernutrition, were found to be prevalent in women desiring to pregnancy within 1 year, and the risks were significantly more in women from tribal area.

## Data Availability

Data will be available for research on reasonable request to the corresponding author.

## Abbreviations

PCC: Preconception care
PHCs: Primary Health Centers
ASHAs: Accredited Social Health Activists
WHO: World Health Organization
BMI: Body mass index
SPSS: Statistical Package for Social Sciences
RPMNCHA: Reproductive, Preconception, Maternal Neonatal Child Health and Adolescents
